# Spotlight on early-career researchers: an interview with Jelena Baranovic

**DOI:** 10.1038/s42003-018-0173-9

**Published:** 2018-11-14

## Abstract

Jelena Baranovic began her independent career at University of Edinburgh in September 2018. In this short Q&A she tells us about her experience as an early career researcher, the advice she would give to her younger self, and the lessons learned from studying ion channel physiology, for both biology and career development.

**Figure:**
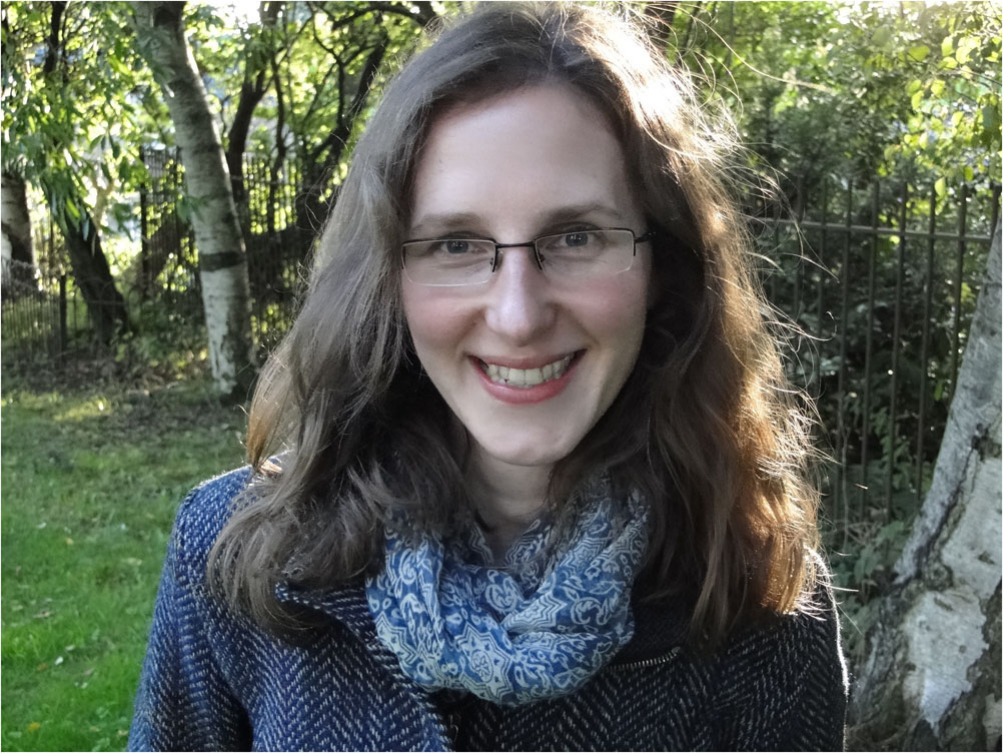
Image credit: Jelena Baranovic

Please tell us about your research interests.

My research focuses on a particular class of proteins from our brains known as glutamate gated ion channels. These neuroreceptors are ubiquitous in the central nervous system, where they mediate communication between neurons. Their ability to rapidly transmit signals between neurons is essential for healthy neuronal development and for many cognitive processes, such as learning and memory. Unsurprisingly, their dysfunction has been implicated in various neurological disorders, including epilepsy and neurodegenerative diseases. I use various biophysical techniques to study the working principles of these important receptors, at the single-molecule and macroscopic level. The aim is to understand how the behaviour and modulation of these receptors enables neuronal communication at the molecular level in the highly dynamic environment of the synapse.

What has your journey been to this point?

I obtained my undergraduate degree in molecular biology at the University of Zagreb and my bachelor thesis was a collaboration between Zagreb University and the University of Oxford, where I worked with Dr. Teuta Pilizota on FoF1-ATPase. This was a short but formative experience without which I am not sure I would be working in science today. It was also the point where I realized I wanted to use biophysical approaches to address biological questions. They reveal a more quantitative side of biology that is very satisfying to me.

I then came back to Oxford to pursue a PhD in the group of Prof. John F. Ryan, where I first started working with glutamate gated ion channels using atomic force microscopy and bilayer recordings. Glutamate receptors can be challenging to work with but I found it rewarding to investigate proteins that play such an essential role in our brain. After my PhD I decided to learn patch clamp electrophysiology—another technique with which I could study these receptors—and that led me to my postdoc in the group of Prof. Andrew Plested at the Leibniz Institute for Molecular Pharmacology in Berlin.

Postdoc years can be quite…existential, in many ways, so I still cannot believe I had such an overwhelmingly positive and encouraging experience. Thanks to that, I now have a very good idea about how life in a good research group looks like and, hopefully, this will help me establish my own group at the University of Edinburgh, UK, where I started this past September.

I have yet to figure out how to juggle research and teaching, but I would like to develop a collaborative atmosphere in the lab where team members feel that we are all working together towards common goals. This might seem obvious in a research lab, but I personally think that it is only achieved by targeted effort. Transparency and decision making at a group level are important tools in creating such a work environment, and I have recently come across the idea of a lab manual^1^—just in time to start writing one.

The journey—and it is all about the journey—is effectively a list of people and places that provided me with ample opportunities for scientific growth and continuous support. Certainly for me, these factors are crucial for doing research and attracting young talent.

What are your predictions for your field in the near future?

When we study these proteins, we often have to use conditions that are very different from their natural environment. This is slowly changing thanks to various technological advancements. For example, structural studies were, for decades, limited to only specific domains of these proteins as the full-length form was extremely challenging to work with. Today, technological breakthroughs in cryo-electron microscopy are slowly making it a norm to study full-length structures of various ion channels, rather than just their soluble domains. We also know that these proteins are a part of large supramolecular assemblies in vivo and it is becoming more common to study them in association with their closely interacting partners. I am sure we will also be seeing further developments in different super-resolution imaging techniques allowing us to observe glutamate receptors and other ion channels as they go about their normal life in the membrane. In case of glutamate receptors, this will reveal the dynamic network of protein interactions underlying synaptic transmission. This is essential for our understanding of the neuronal communication in health and disease.

From a more mechanistic point of view, there are methods which allow us to study either structure or activity of ion channels at very high resolution. While we can computationally simulate structural changes in these proteins at different stages of their activation cycle, the experimental techniques to simultaneously observe changes in structure and function of ion channels at high resolution are somewhat lacking. Some techniques, however, such as serial femtosecond crystallography (using X-ray free electron lasers), hold great promise in that respect. But I probably overstepped into the not-so-near-future here…

Can you speak of any challenges that you have overcome?

I have a tendency to wait until “everything is ready” before making the final step. I am not suggesting rushing things is good, but especially when it comes to applying for independent positions, postponing it for too long could backfire. There were many things in my postdoc that I thought should be done before I started applying for the next step. My postdoc was a steep learning curve and made me grow tremendously as a scientist. But there comes a point where the growth curve saturates and, if research is what one wants to pursue, then I would say this is a natural point to seek something more independent, even if there are some experiments still left to be done.

Also, I am not particularly loud by nature and speaking in public used to terrify me. Not that one has to be loud to make it in science, but it is important to be able to articulate questions, opinions, and results in a more public setting. At some point I just decided to say yes to any public-speaking opportunity that came my way. The frequent exposure definitely helped and it allowed me to get to know the people in the field interested in my area of research. That said, I still have work to do to overcome all of these challenges, but there have definitely been some improvements over the years.

What advice would you give to your younger self?

To get out there a bit more. I don’t think of it as “professional networking”, but just talking to people whose work you are naturally interested in and with whom you share professional and often other interests and passions. Also, have a senior or more established colleague to introduce or advocate for you. That will help a great deal.

And to read more. But this is also advice to my present and future self.

What life lesson have you learned from studying ion channel physiology?

The lessons are numerous and the list continues to grow! But the important ones are: that patience pays off, sometimes; and that not much happens without auxiliary proteins—it is invaluable to have somebody to share your ideas and ramblings with and it helps a lot to stay motivated.

*This interview was conducted by Associate Editor Yomayra Guzmán*
